# Supplementary material for: Local Control of a Single Nitrogen-Vacancy Center by Nanoscale Engineered Magnetic Domain Wall Motion
Source: ACS Nano. 2023 Dec 5;17(24):25689–96. doi: 10.1021/acsnano.3c10633 (PMC10753891; doi:10.1021/acsnano.3c10633)
Supplement: Supplementary file 1 — nn3c10633_si_001.pdf [file nn3c10633_si_001.pdf]

Supporting Information

Local Control of a Single Nitrogen-Vacancy Center by Nanoscale Engineered Magnetic Domain Wall Motion

Nathan J. McLaughlin[1], Senlei Li[2], Jeffrey A. Brock[3], Shu Zhang[4], Hanyi Lu[1], Mengqi Huang[2], Yuxuan Xiao[3], Jingcheng Zhou[2], Yaroslav Tserkovnyak[5], Eric E. Fullerton[3], Hailong Wang[2], and Chunhui Rita Du[1,2,*]

[1]Department of Physics, University of California, San Diego, La Jolla, California 92093, USA
[2]School of Physics, Georgia Institute of Technology, Atlanta, Georgia 30332, USA
[3]Center for Memory and Recording Research, University of California, San Diego, La Jolla, California 92093-0401, USA
[4]Max Planck Institute for the Physics of Complex Systems, Dresden 01187, Germany
[5]Department of Physics and Astronomy, University of California, Los Angeles, California 90095, USA

*Correspondence to: cdu71@gatech.edu

1. Sample information and magneto-transport characterizations

Co-Ni-based multilayer heterostructures were prepared in a sputtering chamber with the based pressure below 2.0 $\times 10^{-8}$ Torr. The individual deposition rates of Co, Ni, Pt, and Ta were calibrated to be 0.21 Å/s, 0.27 Å/s, 0.59 Å/s, and 0.28 Å/s, respectively.[1] Prepared Co-Ni multilayer samples show spontaneous perpendicular magnetic anisotropy. Figures S1A-S1C show field dependent magnetization of substrate/Pt(5)/$[Co(0.5)Ni(0.5)]_1$/Ta(5), substrate/Pt(5)/$[Co(0.5)Ni(0.5)]_2$/Ta(5), and substrate/Pt(5)/$[Co(0.5)Ni(0.5)]_3$/Ta(5) samples measured at room temperature. Numbers in the brackets indicate the thickness of each layer in nanometers. The external magnetic field B_{ext} is applied perpendicular to the sample plane and the saturation magnetization is characterized to be ~6250 Gauss (G), ~6763 G, and ~7813 G for samples with one, two, and three repetitions of the Co-Ni layer. In the present work, we focus on $[Co\text{-}Ni]_2$ samples to study spin current-driven domain wall motion and local control of nitrogen-vacancy (NV) spin properties. $[Co\text{-}Ni]_1$ and $[Co\text{-}Ni]_3$ are utilized as control samples to investigate the internal structure of formed magnetic domains.[2–4]

We patterned the Co-Ni multilayer films into Hall cross devices for spin-orbit-torque (SOT)-driven deterministic magnetic switching measurements. The electrical measurement sequence consists of a write current pulse with a time duration of ~30 milliseconds followed by a read current pulse to record the anomalous Hall voltage of the Co-Ni device. The time interval between subsequent write and read current pulses is ~2 seconds to minimize the effects of Joule heating. Figures S2A and S2B show two typical sets of SOT-driven magnetic switching measurement results of the $[Co\text{-}Ni]_2$ device. The measured anomalous Hall signals exhibit positive (negative) jumps above the negative (positive) critical write currents. The magnetic switching curves change their polarity when reversing the direction of the longitudinal bias magnetic field, which is consistent with the mechanism of SOT-driven deterministic magnetic switching.[5] Compared to the variation of the Hall signal in the field-induced switching measurements, the current-induced switching experiments achieve a 70% switching efficiency for $[Co\text{-}Ni]_2$ devices.

2. Relevant NV spin physics and characterizations of NV-to-sample distance

To ensure single-spin sensitivity and nanoscale spatial resolution, we utilized diamond cantilevers containing single, optically addressable NV centers[6,7] to perform scanning NV magnetometry measurements. Figure S3A shows a typical confocal image of a diamond cantilever containing an NV single electron spin. Figure S3B shows the NV energy levels as a function of external magnetic field B_{ext} applied along the NV spin orientation. When $B_{ext} > 0$, the Zeeman effect lifts the degenerancy of the NV spin energies and separates the $m_s = -1$ and $m_s = +1$ spin states by an energy gap of $2\gamma B_{ext}$, where γ is the gyromagnetic ratio of NV centers. This three-level spin system can be optically accessed by spin-dependent photoluminescence (PL), where the $m_s = \pm 1$ NV spin states are more likely to be trapped by a non-radiative pathway through an intersystem crossing and back to the $m_s = 0$ ground state, emitting reduced PL. Figure S3C shows a typical set of NV electron spin resonance (ESR) spectrum with a pair of split NV spin energies. The magnitude of the local static magnetic field B_{NV} that is parallel to the NV spin orientation $\mathbf{e_{NV}}$ can be obtained as follows:[7]

$$B_{\mathrm{NV}} = \pi(f_+ - f_-)/\gamma \tag{1}$$

where f_+ and f_- correspond to the upper and lower NV ESR energies. In our measurements, B_{NV} contains contributions from both the external magnetic field B_{ext} and the magnetic stray field B_{F} arising from the magnetic device:

$$B_{\mathrm{NV}} = B_{\mathrm{ext}} + B_{\mathrm{F}} \tag{2}$$

By subtracting B_{ext}, which can be precisely characterized by NV measurements, from B_{NV}, the static stray field B_{F} produced by the magnetic sample can be quantitatively obtained. By scanning the NV center over a mesoscopic length scale above the sample surface, we can map spatially varying B_{F} as shown in Figure 1E in the main text.

The spatial resolution of the presented scanning NV measurements is primarily determined by the NV-to-sample distance. Next, we present the detailed method to characterize this important instrumental parameter. Figure S4A shows the schematic of our measurement platform and the coordinate system used for numerical analysis. We assume that the Co-Ni magnetic device occupies the space Ω with $z \leq 0$ with its top surface lying in the x-y plane ($z = 0$). θ and φ represent the polar and azimuthal angles characterizing the NV spin orientation $\mathbf{e}_{\mathbf{NV}}$, which are equal to 54 and 0 degree in the current measurement configuration. NV center is located in the scanning plane of $z = d$. Quantitative measurement of the nanoscale NV-to-sample distance d can be realized by mapping the spatially dependent magnetic stray field B_{F} arising from the patterned Co-Ni multilayer device. Figure S4B shows a typical set of one-dimensional B_{F} data measured along the x-axis across the edge of the Co-Ni Hall device. Note that the presented scanning measurement was performed in a "contact" mode, where the diamond cantilever is attached to the surface of sample with a stand-off distance to be zero. According to dipole interactions, $B_{\mathrm{F}}(x)$ can be expressed by the following equation:[8]

$$B_{\mathrm{F}}(x) = \left[\mathbf{B}^{\mathbf{edge}}\left(x - \frac{w_{\mathrm{c}}}{2}\right) - \mathbf{B}^{\mathbf{edge}}\left(x + \frac{w_{\mathrm{c}}}{2}\right)\right] \cdot \mathbf{e}_{\mathbf{NV}} \tag{3}$$

where w_{c} is the width of the Hall device and $\mathbf{B}^{\mathbf{edge}}(x)$ is a vector field with the x, y, z components to be:[8]

$$\begin{cases} B_x^{\mathrm{edge}}(x) = 2M_s t_{\mathrm{F}} \dfrac{d + \Delta d(x)}{x^2 + [d + \Delta d(x)]^2} \\ B_y^{\mathrm{edge}}(x) = 0 \\ B_z^{\mathrm{edge}}(x) = -2M_s t_{\mathrm{F}} \dfrac{x}{x^2 + [d + \Delta d(x)]^2} \end{cases} \tag{4}$$

Here, M_s and t_{F} are the saturation magnetization and thickness of the Co-Ni sample, respectively, and Δd characterizes the variation of the sample height which is simultaneously recorded by atomic force microscopy during the scanning measurements. By fitting $B_{\mathrm{F}}(x)$ to Eq. (3), the NV-to-sample distance d is extracted to be 58.7 ± 3.6 nm. In the current studies,

the NV-to-sample distance was ~59 nm for scanning measurements of NV photoluminescence (Figure 4 in the main text) to enhance the signal-to-noise ratio. For magnetic stray field imaging in Figures 1-3 and Figure S6, we increased the NV-to-sample distance to 159 nm with a probe height of 100 nm to improve the measurement quality.

3. Reconstruction of static magnetization patterns

By performing scanning NV measurements, we obtained a series of stray field B_F maps of the Co-Ni multilayer device as shown in Figures 2A-2H in the main text. In this section, we present the detailed method to reconstruct the magnetization patterns of the Co-Ni device from the obtained stray field maps. Generally, the stray field distribution $\mathbf{B}_F(\mathbf{R})$ of a magnetic system is fundamentally determined by its static magnetization distribution $\mathbf{M}(\mathbf{R}')$ as follows:[9]

$$\mathbf{B}_{\mathrm{F}}(\mathbf{R}) = \int d^3\mathbf{R}' \mathcal{D}(\mathbf{R},\mathbf{R}')\mathbf{M}(\mathbf{R}'), \tag{5}$$

where $\mathcal{D}(\mathbf{R},\mathbf{R}') = -\nabla_{\mathbf{R}}\nabla_{\mathbf{R}'}\left(1/|\mathbf{R}-\mathbf{R}'|\right)$ is the magnetostatic Green's function tensor between coordinates $\mathbf{R} = (x, y, z)$ and $\mathbf{R}' = (x', y', z')$. Focusing on the magnetic field at the position of the NV center ($z = d$), we take the Fourier transform in the x and y directions, where translational symmetries are present with $\mathbf{r} = (x, y)$ and $\mathbf{k} = (k_x, k_y)$:

$$\mathbf{B}_{\mathrm{F}}(\mathbf{k}) = \int \mathbf{B}_{\mathrm{F}}(\mathbf{r}, d) e^{i\mathbf{k}\cdot\mathbf{r}} d^2\mathbf{r},$$

$$\mathbf{M}(\mathbf{k}, z) = \int \mathbf{M}(\mathbf{r}, z) e^{i\mathbf{k}\cdot\mathbf{r}} d^2\mathbf{r}. \tag{6}$$

Similarly, we can obtain the Green's function tensor in the Fourier space for $\alpha, \beta = x, y$:[9]

$$\begin{cases} D_{\alpha\beta}(\mathbf{k}, z') = -2\pi\left(k_\alpha k_\beta / k\right) e^{-k(d-z')}, \\ D_{\alpha z}(\mathbf{k}, z') = -2\pi i k_\alpha e^{-k(d-z')}, \\ D_{zz}(\mathbf{k}, z') = 2\pi k e^{-k(d-z')}. \end{cases} \tag{7}$$

where $k = |\mathbf{k}|$, thus

$$\mathbf{B}_{\mathrm{F}}(\mathbf{k}) = \int D(\mathbf{k}, z')\mathbf{M}(\mathbf{k}, z') dz' \ . \tag{8}$$

Due to the perpendicular anisotropy, the magnetization of the Co-Ni device is spontaneously aligned along the z-axis direction: $\mathbf{M}(\mathbf{k}, z) = M(\mathbf{k})\hat{\mathbf{z}}$. Here, we have assumed a uniform magnetization distribution along the thickness direction of the atomically thin Co-Ni sample. Next, we focus on the component of the magnetic stray field along the NV axis B_F generated by the static perpendicular magnetization of the sample:

$$B_{\mathrm{F}}(\mathbf{k}) = \int_{-t_{\mathrm{F}}}^{0} \mathbf{e}_{\mathrm{NV}} \cdot D(\mathbf{k}, z') \cdot M(\mathbf{k})\hat{\mathbf{z}}dz'$$
$$= 2\pi e^{-kd}\left(1-e^{-kt_{\mathrm{F}}}\right)\left(\cos\theta - i\frac{k_x}{k}\sin\theta\cos\varphi - i\frac{k_y}{k}\sin\theta\sin\varphi\right)M(\mathbf{k}), \tag{9}$$

where t_{F} is the thickness of the Co-Ni multilayer, θ and φ represent polar and azimuthal angles of the NV spin axis, as illustrated in Figure S4A. Finally, we introduce an inverse Fourier transform on Eq. (9) to reconstruct the magnetization in the real space:

$$M(\mathbf{r}) = \frac{1}{(2\pi)^2}\int M(\mathbf{k})e^{-i\mathbf{k}\cdot\mathbf{r}}d^2\mathbf{k}. \tag{10}$$

Using the method presented above, Figures S5A-S5H show 2D maps of the reconstructed Co-Ni magnetization measured at the corresponding points ("A" to "H") on a current-driven switching loop (Figure S5I), confirming the SOT-driven deterministic magnetic switching behavior.

4. Probing the chirality of formed magnetic domain walls in Co-Ni multilayers

In this section, we present the method to diagnose the internal domain wall structure of Co-Ni multilayer devices by scanning NV magnetometry measurements. We start our discussions by considering a general situation where a domain wall lies along the y-axis direction with its central position located at $x = 0$ as illustrated in Figure S6A. The out-of-plane and in-plane magnetization of the sample along the x-axis direction can be expressed as:[8,10]

$$M_{\perp}(x) = -M_s \tanh(x/\lambda)$$

$$M_{\parallel}(x) = M_s / \cosh(x/\lambda) \tag{11}$$

where M_s is the saturation magnetization and λ is the domain wall width. Assuming the domain wall is infinite along y-axis, the stray field generated by the out-of-plane magnetization component at an NV site (x, $y = 0$, $z = d$) can be expressed as:[8]

$$\begin{cases} B_x^{\perp}(x) \approx \dfrac{4M_s t_{\mathrm{F}} d}{x^2+d^2} \\ B_z^{\perp}(x) \approx -\dfrac{4M_s t_{\mathrm{F}} x}{x^2+d^2} \end{cases} \tag{12}$$

where $B_x^{\perp}$ and $B_z^{\perp}$ characterize the stray field along the x and z axis, respectively, t_{F} is the thickness of the Co-Ni multilayer, and d is the NV-to-sample distance as discussed above. The stray fields arising from the in-plane magnetization can be expressed as:[8]

$$\begin{cases} B_x^{\parallel}(x) \approx 2\pi M_s t_F \lambda \dfrac{x^2 - d^2}{\left(x^2 + d^2\right)^2} \\ B_z^{\parallel}(x) \approx 4\pi M_s t_F \lambda \dfrac{xd}{\left(x^2 + d^2\right)^2} \end{cases} \tag{13}$$

The total stray field incorporating contributions from both in-plane and out-of-plane magnetization can be expressed as:[8]

$$\mathbf{B}_{\mathrm{F}}(x) = \mathbf{B}^{\perp}(x) + \mathbf{B}^{\parallel}(x)\cos\psi \tag{14}$$

where $\mathbf{B}^{\perp}(x) = (B_x^{\perp}, 0, B_z^{\perp})$, $\mathbf{B}^{\parallel}(x) = (B_x^{\parallel}, 0, B_z^{\parallel})$, and ψ is the in-plane angle between the magnetization and the $+x$ axis direction, as illustrated in Figure S6A. Microscopically, $\psi = 0$ and $\psi = \pi$ correspond to the right- and left-handed Néel type domain walls, while $\psi = \pi/2$ characterizes the Bloch type domain wall. Fitting the spatial profile of B_{F} using Eq. (14), and a domain wall width $\lambda = 10$ nm, the in-plane magnetization projection angle ψ of a formed domain wall can be quantitatively obtained. The domain wall width is not a fitting parameter, as it is below our measured spatial resolution. The fitting results do not depend strongly on this value. Figures S6B-S6D show the measured magnetic stray field B_{F} maps generated by [Co-Ni]$_1$, [Co-Ni]$_2$, and [Co-Ni]$_3$ samples. Figures S6E-S6G show one-dimensional B_{F} measured along the linecuts (denoted by the dashed lines) across the formed magnetic domain walls shown in Figures S6B-S6D. By fitting the data to Eq. (14), the in-plane magnetization projection angle ψ is obtained to be 180 $\pm$ 6 degrees, 156 $\pm$ 1 degrees, and 96 $\pm$ 10 degrees for [Co-Ni]$_1$, [Co-Ni]$_2$, and [Co-Ni]$_3$ multilayers, respectively. Although the difference might be minor between different domain wall types, in an intuitive picture, we expect that magnetic domain walls formed in Co-Ni multilayers systematically evolve from the left-handed Néel to the Bloch type structure when increasing the number of the Co-Ni repetition layer from one to three. A comparison between these domain wall types to the fittings to the [Co-Ni]$_1$ and [Co-Ni]$_2$ stray field data are shown in Figures S6J-S6K. The change of the internal structure of the formed magnetic domain walls is fundamentally correlated to the variation of the interfacial Dzyaloshinskii–Moriya interaction in the magnetic heterostructures, in consistent with previous research works.[11–14]

5. NV ESR spectra showing local control of NV photoluminescence and spin level energies

As discussed in the main text, nanoscale engineered domain wall motion modifies the local static field environment of a proximate NV center in a reconfigurable way, enabling effective control of NV photoluminescence and ESR energies. Figures S7A-S7C illustrate SOT-driven magnetic domain wall motions relative to an NV center. In the initial state, the NV center is positioned right above the formed domain wall (Figure S7A). Application of a write current pulse I_{write} of −24 mA under appropriate magnetic field configuration exerts SOT on local Co-Ni magnetization and drives domain wall propagations (Figure S7B) while sending a

current pulse I_{write} of 28 mA will cause retraction of the magnetic domain wall back to the original position (Figure S7C). Figures S7D-S7F present the corresponding ESR spectra recorded at a fixed NV position in the three situations discussed above (Figures S7A-S7C). When the magnetic domain wall is moving to the position right below the NV center, we observe enhanced NV photoluminescence due to a suppressed NV off-axis field arising from the competing effect of the local magnetic stray field and the external bias field (Figures S7D and S7F). When the magnetic domain wall is moving away from the NV center, magnetic stray field arising from the uniform magnetic domain is negligibly small, leading to reduced NV photoluminescence (Figure S7E). The variation of the local static magnetic field at the NV site also causes noticeable changes of the NV ESR frequency. Alternate applications of write current pulses with opposite polarity (I_{write} = 28 mA and I_{write} = −24 mA) will reversibly drive the magnetic domain wall away from or back towards the NV center, resulting in switching of the NV ESR frequencies between two different states as shown in Figure S8. Similar to the NV photoluminescence and coherence time, the variations of the NV ESR frequencies can also be correlated to the measured anomalous Hall signals of the Co-Ni device. We note that the full width at half max of the measured NV ESR peaks is 31.6 MHz and 18.3 MHz, respectively, when the magnetic domain wall is located right below and stays away from the NV center. Such discrepancy is attributed to the variation of the intrinsic NV coherence time, which will be discussed in detail in the following section.

6. Domain wall motion-controlled NV spin coherence time

In this section, we present experimental details and a theoretical model to describe the variation of NV coherence time in response to nanoscale domain wall motion. Figure S9A shows the measurement protocol (Hahn echo sequence) to characterize the NV coherence time. A green laser pulse was first applied to initialize the NV spin to the $m_s = 0$ state followed by a $\pi/2$ microwave pulse with an ESR frequency f_- applied to rotate the NV spin to the equator of the Bloch sphere. After a free evolution time $\tau/2$, a π pulse was then applied followed by another free evolution time $\tau/2$. Lastly, the second $\pi/2$ microwave pulse with a frequency f_- was applied before reading out the final NV spin state by a green laser pulse. Figure S9B shows the measured NV PL intensity as a function of the free evolution time τ under three different experimental conditions. The intrinsic NV coherence time T_2 can be quantitatively extracted by fitting the data to an exponential decay function:[15,16]

$$PL(\tau) = Ae^{-\frac{\tau}{T_2}} + C \qquad (15)$$

where A and C are fitting parameters. When the diamond cantilever containing a single NV center is away from the Co-Ni device, the intrinsic NV quantum coherence time T_2 is measured to be 8.2 μs. When the diamond cantilever is in contact with the Co-Ni device (NV-to-sample distance $d \approx 59$ nm) at a position that is away from the formed magnetic domain wall, the obtained NV T_2 slightly decreases to 7.3 μs. In contrast, when the diamond cantilever is positioned right above a formed domain wall (still in the "contact mode" with $d \approx 59$ nm), the measured NV T_2 shows a dramatic reduction to 0.6 μs. Figure S9C shows a stray field image

of a formed magnetic domain wall in the Co-Ni device. Figures S9D and S9E plot one-dimensional variation of the NV spin decoherence rate ($1/T_2$) and relaxation rate ($1/T_1$) measured along the linecut (dashed lines) shown in Figure S9C. The obtained NV T_2 shows a significant decrease when the diamond cantilever is scanned across the magnetic domain wall.

The observed decrease of NV T_2 is attributed to the gapless spin excitations arising from the magnetic domain wall with a divergent susceptibility in the zero-frequency limit.[17–20] When the NV center is away from the magnetic domain wall and being positioned above a uniform magnetic domain where the Co-Ni magnetization is perpendicularly aligned, the out-of-plane magnetic anisotropy significantly raises the minimal magnon excitation energy, resulting in a negligible effect on the NV coherence time.[21,22] When the NV center is positioned near the domain wall, our measured relaxation time T_1 is not modified either, because, at the currently used sensor to sample distance and ESR frequency, the NV center does resonantly pick up the noise from the gapless mode in the domain wall. Here, we note that other factors such as the external bias field angle would be a secondary factor in affecting the NV quantum coherence time, which is not included in our theoretical model presented below.

Next, we theoretically discuss the magnetic noise in the presence of a string-like domain wall. Firstly, the scattering of the bulk spin waves off the domain wall may modify their spectra[23] and diffusion properties, which can contribute to the subgap magnetic noise[24,25] differently from the bulk. More strikingly, a new spin-wave mode arises on the domain wall, analogous to an elastic wave propagating on a string, which is a gapless mode in an ideal model ignoring pinning effects. This mode is the main object of consideration here as it dominates the contribution to the decoherence rate of the NV center in proximity. To see this, we first recapitulate the general expressions of the relaxation and coherence time of the NV center, which can be treated as a two level system with the following Hamiltonian:[19]

$$H = \frac{\Delta}{2}\sigma^z + \sigma^z \otimes \mathcal{X} + \sigma^+ \otimes \mathcal{Y} + \sigma^- \otimes \mathcal{Y}^\dagger, \quad (16)$$

where Δ is the NV ESR frequency, $\sigma^\pm = \sigma^x \pm i\sigma^y$, and $\sigma^{x,y,z}$ are Pauli matrix. The operators $\mathcal{X}$ (Hermitian) and $\mathcal{Y}$ describe the longitudinal and transverse interaction between the NV spin and Co-Ni multilayer. Treating $\mathcal{X}$ and $\mathcal{Y}$ as perturbations, the NV spin relaxation time T_1 and the spin coherence time T_2 can be extracted under the Born-Markovian approximation:[19,26]

$$T_1^{-1} = S_{\mathcal{Y}}(\omega = \Delta), \text{ and } T_2^{-1} = \frac{1}{2}T_1^{-1} + S_{\mathcal{X}}(\omega = 0), \quad (17)$$

where the power spectrum of operators $\mathcal{X}$ and $\mathcal{Y}$ is defined as: $S_{\mathcal{X}}(\omega) = \int dt e^{i\omega t} \left\langle \left\{ \mathcal{X}^\dagger(t), \mathcal{X} \right\} \right\rangle$ and $S_{\mathcal{Y}}(\omega) = \int dt e^{i\omega t} \left\langle \left\{ \mathcal{Y}^\dagger(t), \mathcal{Y} \right\} \right\rangle$. We thus expect the decoherence rate $1/T_2$ to pick up an extra contribution when there exists a zero-frequency mode in the material, supposing it generates a

magnetic field along the NV axis. We next turn to the modeling of the domain wall to show that it hosts such a zero mode.

A string-like domain wall in a thin film can be described by two parameters: its position $X(y, t)$ and the azimuthal angle $\Phi(y, t)$ of spins on the domain wall. Figure S10A shows the coordinate system used for numerical analysis. Following Ref. 19 and Ref. 26, we derive the equation of motion for the dynamics of a smooth domain-wall string with the unit vector of magnetization $\mathbf{m} \equiv \mathbf{M}/M$ interpolating between $\mathbf{m}(y) = (0, 0, +1)$ and $(0, 0, -1)$. The unit vector $\mathbf{m} = (\sin\vartheta \cos\phi, \sin\vartheta \sin\phi, \cos\vartheta)$ is parametrized in terms of its polar and azimuthal angles ϑ and ϕ, as shown in Figure S10A. We consider the magnetic potential energy of the system assuming the domain wall is nearly straight and uniform along the y axis. It consists of the exchange energy:

$$U_{\text{exchange}} = At_F \int d^2\mathbf{r} \partial_i \mathbf{m} \cdot \partial_i \mathbf{m} = At_F \ell \int dx \left[(\partial_x \vartheta)^2 + \sin^2 \vartheta (\partial_x \phi)^2 \right], \tag{18}$$

where A is the exchange stiffness, t_F is the film thickness, and ℓ is the domain-wall length, the energy of anisotropy:

$$U_{\text{anisotropy}} = -Kt_F \int d^2\mathbf{r} m_z^2 = -Kt_F \ell \int dx \cos^2 \vartheta, \tag{19}$$

the dipolar energy considering the magnetic field from magnetic charge accumulation $\nabla \cdot \mathbf{M}$ at the film surfaces and the domain wall:

$$U_{\text{dipolar}} \approx 2\pi t_F \int d^2\mathbf{r} \left(M_z^2 + M_x^2 \right) = 2\pi M^2 t_F \ell \int dx \left(\cos^2 \vartheta + \sin^2 \vartheta \cos^2 \phi \right), \tag{20}$$

where the first term weakens the easy-axis anisotropy in the bulk and the second term favors the Bloch type of domain wall configuration, note that the dipolar energy here is approximated to a local anisotropy form assuming that the film is thin and the domain wall is narrow $t_F \sim \lambda \ll \ell$. and the interfacial Dzyaloshinskii-Moriya interaction:

$$U_{\text{DMI}} = Dt_F \int d^2\mathbf{r} \left(m_z \nabla \cdot \mathbf{m} - \mathbf{m} \cdot \nabla m_z \right) = Dt_F \ell \int dx \left(\cos\phi \, \partial_x \vartheta - \frac{1}{2} \sin 2\vartheta \sin\phi \, \partial_x \phi \right), \tag{21}$$

which favors instead the Néel type. Minimizing the total potential energy using the conventional domain wall solution $\cos\vartheta(x) = -\tanh[(x - X)/\lambda]$ and $\phi(x) = \Phi_0$ with respect to parameters λ (the characteristic domain-wall width) and Φ_0 yields:

$$\lambda = \sqrt{\frac{A}{K - 2\pi M^2 \sin^2 \Phi_0}} \quad \text{and} \quad \cos\Phi_0 = \frac{D}{8M^2 \lambda}. \tag{22}$$

Notably, for $|D| < 8M^2\lambda$, the competition between the magnetic dipolar interaction and DMI stabilizes a domain wall with an intermediate state between the Bloch and Néel type. We quote

the material parameters from Ref. 1: exchange stiffness $A \sim 10$ pJ/m, easy-axis anisotropy $K \sim 1.1\times10^6$ J/m^3, saturated magnetization $4\pi M = 6763$ G, and DMI parameter $D \sim -0.63$ mJ/m^2, which yields solutions $\lambda \sim 3.0$ nm and $\Phi_0 \sim 155$ degrees, in agreement with the experimentally fitted configuration.

The total potential energy proportional to the length of the domain wall thus defines a tension for the domain-wall string $\sigma = \left[\left(2A - \pi D^2/8M^2\right)/\lambda + 2K_{\text{eff}}\lambda\right]t_F$, where $K_{\text{eff}} = K - 2\pi M^2[1-(D/8M^2\lambda)^2]$. For small deviations from this configuration, the energy varies quadratically in $\delta\Phi = \Phi - \Phi_0$, assuming the domain-wall width λ stays invariant as an approximation in the highly nonlinear Eq. (22), and provides a stiffness $\kappa = (8\pi M^2\lambda - \pi D^2/16M^2\lambda)t_F$ for misalignment between the in-plane magnetization of the domain wall and the preferred azimuthal angle Φ_0. Noting that the azimuthal angle Φ is defined with respect to the domain wall direction, we have the Lagrangian for the domain wall with slowly varying $X(y, t)$ and $\Phi(y, t)$:[18,27]

$$\mathcal{L} = g\frac{\partial X}{\partial t}\Phi - \frac{\sigma}{2}\left(\frac{\partial X}{\partial y}\right)^2 - \frac{\kappa}{2}\left(\Phi - \Phi_0 - \frac{\partial X}{\partial y}\right)^2. \tag{23}$$

The first term represents a gyroscopic coupling of strength $g = -2t_F M/\gamma_M$ between the position of the domain wall X and its azimuthal angle Φ,[28] which is rooted in the topological nature of the domain wall. Here γ_M is the gyromagnetic ratio of the Co-Ni film.

Minimization of the action $S = \int_\Omega dtdy\mathcal{L}$ with respect to the magnetization angle Φ yields

$$\Phi = \Phi_0 + \frac{g}{\kappa}\frac{\partial X}{\partial t} + \frac{\partial X}{\partial y}, \tag{24}$$

and eliminating Φ yields a Lagrangian for the field $X(y, t)$,

$$\mathcal{L} = \frac{\rho}{2}\left(\frac{\partial X}{\partial t}\right)^2 - \frac{\sigma}{2}\left(\frac{\partial X}{\partial y}\right)^2 + g\frac{\partial X}{\partial t}\frac{\partial X}{\partial y}, \tag{25}$$

with the Döring mass density $\rho = g^2/\kappa$. This leads to the following equation for the domain wall dynamics:

$$\rho\frac{\partial^2 X}{\partial t^2} - \sigma\frac{\partial^2 X}{\partial y^2} + 2g\frac{\partial^2 X}{\partial t\partial y} = 0, \tag{26}$$

From this equation of motion, the spin waves propagating along the domain wall are gapless, with a dispersion relation:

$$\omega = v_{\pm}k, \quad v_{\pm} = \frac{g}{\rho} \pm \sqrt{\frac{g^2}{\rho^2} + \frac{\sigma}{\rho}}. \tag{27}$$

where k is the momentum along the domain wall (Fourier transform in the y direction). Using the same material parameters mentioned above, plus the film thickness $t_F \sim 1$ nm, and the gyromagnetic ratio $\gamma_M \sim 2\pi \times 28$ GHz/T, we obtain an estimation of the gyrotropic coupling $g \sim 6.1 \times 10^{-15}$ Kg/s, the line tension $\sigma \sim 1.1 \times 10^{-11}$ J/m, the in-plane stiffness $\kappa \sim 1.3 \times 10^{-12}$ J/m, and the mass density $\rho \sim 2.9 \times 10^{-17}$ kg/m. The domain wall hosts a zero mode in the limit of $k \to 0$, $\omega \to 0$, corresponding to a translation of the position of the domain wall, which does not cost magnetic energy of the system. While deviations from the linear dispersion arise at large k due to quadratic corrections in the bending energy of the domain wall, this consideration suffices for our discussion on the low-frequency magnetic noise.

At low frequencies where the bulk spin-wave modes are off resonance with the NV frequency, this gapless mode has the largest contribution to the NV relaxation and decoherence. Since a change in the domain wall position $X(y, t)$ directly changes the total magnetization M_z of the system, the spin susceptibility associated with this mode can be expressed as:

$$\chi_{zz}(k,\omega) = \frac{4\mathcal{S}^2 t_F^{\,2} / \rho}{(\omega - v_+ k)(\omega - v_- k) - i\omega / \tau_s}, \tag{28}$$

where k is the momentum along the domain wall, $\mathcal{S} = 2M / \hbar\gamma$ is the spin density per volume, and we take into account the dissipation by inducing a relaxation time $\tau_s = \lambda\rho / \hbar\alpha\mathcal{S}t_F$, with α the Gilbert damping. Note that this susceptibility is divergent in the ω, $k \to 0$ limit. This is physical as the domain wall can be driven into motion by an arbitrarily small out-of-plane magnetic field to enlarge the domain favored by the field until it occupies the entire material assuming no pinning effect. The spin correlation function S_{zz} is related to the imaginary part of the spin susceptibility χ_{zz}'' by the fluctuation-dissipation theorem, for frequencies much lower than the temperature:[29]

$$\begin{aligned} S_{zz}(k,\omega) &= \frac{4k_B T}{\omega}\chi_{zz}''(k,\omega) \\ &= \frac{16k_B T\mathcal{S}^2 t_F^{\,2} / \rho\tau_s}{(\omega - v_+ k)^2(\omega - v_- k)^2 + \omega^2 / \tau_s^{\,2}}. \end{aligned} \tag{29}$$

where k_B is the Boltzmann constant. As we are only concerned with the magnetic field fluctuations generated by the zero mode, we simplify the model of the domain wall as an infinitely long string sitting at $x = z = 0$ along the y axis, assuming the film thickness t_F and the

domain wall width λ are both very small. The magnetic stray field at the position of the NV center $(x, 0, d)$ generated by the z component of a fluctuation of the spin density $s_z(y, t)$ on the domain wall is given by the magnetostatic relation. As discussed above, we are concerned with the field component B_n along the NV axis $\hat{\mathbf{n}} = (\sin\theta\cos\varphi, \sin\theta\sin\varphi, \cos\theta)$ only,

$$B_n(t;x,d) = 2\pi\hbar\gamma_{\mathrm{M}} \int dy \frac{3(\hat{\mathbf{z}}\cdot\hat{\mathbf{r}})(\hat{\mathbf{n}}\cdot\hat{\mathbf{r}}) - \hat{\mathbf{n}}\cdot\hat{\mathbf{z}}}{r^3} s_z(y,t), \tag{30}$$

where $\mathbf{r} = (x, -y, d)$ is the vector pointing from a point on the domain wall to the location of the NV center. The fluctuation of field $B_n(t; x, d)$ can then be calculated via the spin correlation function:

$$\begin{aligned} S_B(\omega;x,d) &= \int dt e^{i\omega t} \langle B_n(t;x,d) B_n(0;x,d) \rangle \\ &= (2\pi\hbar\gamma_{\mathrm{M}})^2 \int \frac{dk}{2\pi} \mathcal{G}(-k;x,d)\mathcal{G}(k;x,d) S_{zz}(k,\omega), \end{aligned} \tag{31}$$

Here, $\mathcal{G}(k;x,d)$ is the one-dimensional Fourier transform of the magnetostatic relation:

$$\begin{aligned} \mathcal{G}(k;x,d) &= \int dy e^{iky} [3(\hat{\mathbf{z}}\cdot\hat{\mathbf{r}})(\hat{\mathbf{n}}\cdot\hat{\mathbf{r}}) - \hat{\mathbf{n}}\cdot\hat{\mathbf{z}}]/r^3 \\ &= -\frac{1}{3(x^2+d^2)} \left\{ 4\cos\theta G_{1,3}^{2,1}\left[\frac{1}{4}k^2(x^2+d^2) \middle| \begin{matrix} -1/2 \\ 0,1,1/2 \end{matrix} \right] \right. \\ &\quad + 2k^2\left[(x^2-2d^2)\cos\theta - 3dx\sin\theta\cos\varphi \right] K_2\left(|k|\sqrt{x^2+d^2} \right) \\ &\quad \left. + 6id\,\mathrm{sgn}(k) k^2 \sin\theta\sin\varphi\sqrt{x^2+d^2} K_1\left(|k|\sqrt{x^2+d^2} \right) \right\} \end{aligned} \tag{32}$$

where $G_{m,n}^{p,q}\left[\cdot \middle| \begin{matrix} a_1,\ldots,a_p \\ b_1,\ldots,b_q \end{matrix} \right]$ is the Meijer G function, and K_n is the modified Bessel function of the second kind. The second term of the decoherence rate in Eq. (17) can then be calculated by setting $\mathcal{X} = \gamma B_n/2$, $S_{\mathcal{X}}(\omega=0) = (\gamma/2)^2 S_B(\omega=0;x,d)$, where γ is the gyromagnetic ratio of NV centers. As an estimation of the order of the magnitude of the NV decoherence rate,

$$T_2^{-1} \sim (4\pi\hbar)^2 \gamma^2 \gamma_M{}^2 \frac{k_B T\alpha\hbar S^3 t_F{}^3}{\rho^2 v^4 \lambda} \frac{\Lambda^3}{d^4} \sim \frac{(4\pi)^2 \mu_B}{\hbar} \frac{k_B T\alpha M^3 t_{\mathrm{F}}{}^3}{\rho^2 v^4 d\lambda} \left(\frac{\Lambda}{d} \right)^3, \tag{33}$$

Where $\hbar$ = 1.05×10^{-34} Js, Bohr magneton μ_{B} = 9.27×10^{-24} J/T, room temperature T = 300 K, Boltzmann constant k_{B} = 1.38 × 10^{-23} J/K, the spin wave velocity $v_+ \sim v_- \sim v \sim$ 650 m/s, the Gilbert damping $\alpha \sim 0.1$, the NV-to-sample distance $d \sim$ 60 nm, and the film thickness $t_{\mathrm{F}} \sim$ 1 nm. We model the pinning effect of the domain wall here by inducing a lower bound $1/\Lambda$ for

the integral over the wave vector, which defines a soft gap for the domain-wall mode and is necessary to treat infrared divergence. Here, Λ can be perceived as a characteristic length of the domain wall sections set by the pinning sites. Taking $\Lambda \sim 150$ nm, the estimation above yields $1/T_2 \sim 4.8$ MHz, which is of the same order of magnitudes as the measured peak value as shown in Figure S9D. Note that this is the peak value for an NV positioned precisely above the domain wall. However, due to thermal fluctuations, the domain wall position is not well defined. An estimation of the instantaneous fluctuation of the domain wall position with the same momentum cutoff gives $\sqrt{X^2} \sim \left(\pi k_B T \Lambda / \rho v^2\right)^{1/2} \sim 16$ nm, which may broaden the peak and slightly reduce the peak value.

The magnetic noise generated by a local coherent section of the domain wall falls off on a length scale set by the NV distance d roughly as $1/(x^2+d^2)^2$, as given by the magnetostatic relation.[9] Magnetic noise generated by incoherent domain-wall sections can slow down the decay and broaden the measured peak. For those sections, the length scale of signal decay is given by $d_n = \sqrt{d^2 + L_n^2}$, where L_n is the lateral distance to the domain wall segment. Summing over both the coherent and incoherent domain-wall sections,

$$T_2^{-1}(x) \propto \frac{\Lambda_0^{\ 3}}{\left(x^2+d^2\right)^2} + \sum_{n=1}^{n_{\max}} \frac{\Lambda_n^{\ 3}}{\left(x^2+d_n^2\right)^2}, \tag{34}$$

where Λ_n is the segment length for each domain-wall section, and the functional form of $T_2^{-1}(x)$ fits better to one with a slower decay $\propto 1/(x^2+d_{eff}^2)$. This is in agreement with the observation that the peak width of the decoherence rate can be comparable to or exceed that of the stray field B_F: the former characterizes the fluctuations where incoherent contributions need to be taken into account, while the latter reflects the averaged profile. In Figure S10B, we fit the spatially dependent variation of the NV decoherence rate T_2^{-1} across a magnetic domain wall to Eq. (34), from which the effective width d_{eff} is extracted to be 388 nm.

Finally, we remark that the noise generated by the in-plane component of the spin density at the domain wall can be neglected for two reasons. First, the spin susceptibility under an in-plane magnetic field is not divergent in the zero-frequency limit. As detailed in Ref. 18, the time derivative of the in-plane field, rather than the field itself, acts like a force on the domain wall, yielding an extra ω factor in the response function. Secondly, the magnetostatic relation for in-plane components are higher orders in the NV distance. This can be seen from Eq. (13). Our approach of obtaining the relation between the fluctuation of the magnetic field and the fluctuation of spin density here is equivalent to taking a linearized dependence of the stray field B_F on the domain wall position.

References

(1) Brock, J. A.; Kitcher, M. D.; Vallobra, P.; Medapalli, R.; Li, M. P.; De Graef, M.; Riley, G. A.; Nembach, H. T.; Mangin, S.; Sokalski, V.; Fullerton, E. E. Dynamic symmetry breaking in chiral magnetic systems. *Adv. Mater.* **2021**, *33*, 2101524.

(2) Dzyaloshinsky, I. A Thermodynamic theory of “weak” ferromagnetism of antiferromagnetics. *J. Phys. Chem. Solids.* **1958**, *4*, 241.

(3) Moriya, T. Anisotropic superexchange interaction and weak ferromagnetism. *Phys. Rev.* **1960**, *120*, 91.

(4) Fert, A. R. Magnetic and transport properties of metallic multilayers. *Mater. Sci. Forum* **1991**, *59–60*, 439–480.

(5) Liu, L.; Pai, C.-F.; Li, Y.; Tseng, H. W.; Ralph, D. C.; Buhrman, R. A. Spin-torque switching with the giant spin Hall effect of tantalum. *Science* **2012**, *336*, 555.

(6) Doherty, M. W.; Manson, N. B.; Delaney, P.; Jelezko, F.; Wrachtrup, J.; Hollenberg, L. C. L. The nitrogen-vacancy colour centre in diamond. *Phys. Rep.* **2013**, *528* (1), 1–45.

(7) Rondin, L.; Tetienne, J.-P.; Hingant, T.; Roch, J.-F.; Maletinsky, P.; Jacques, V. Magnetometry with nitrogen-vacancy defects in diamond. *Rep. Prog. Phys.* **2014**, 77, 056503.

(8) Tetienne, J.-P.; Hingant, T.; Martínez, L. J.; Rohart, S.; Thiaville, A.; Diez, L. H.; Garcia, K.; Adam, J.-P.; Kim, J.-V.; Roch, J.-F.; Miron, I. M.; Gaudin, G.; Vila, L.; Ocker, B.; Ravelosona, D.; Jacques, V. The nature of domain walls in ultrathin ferromagnets revealed by scanning nanomagnetometry. *Nat. Commun.* **2015**, *6*, 6733.

(9) Guslienko, K. Y.; Slavin, A. N. Magnetostatic Green’s functions for the description of spin waves in finite rectangular magnetic dots and stripes. *J. Magn. Magn. Mater.* **2011**, *323*, 2418–2424.

(10) Wörnle, M. S.; Welter, P.; Giraldo, M.; Lottermoser, T.; Fiebig, M.; Gambardella, P.; Degen, C. L. Coexistence of Bloch and Néel walls in a collinear antiferromagnet. *Phys. Rev. B* **2021**, *103*, 094426.

(11) Shahbazi, K.; Kim, J.-V.; Nembach, H. T.; Shaw, J. M.; Bischof, A.; Rossell, M. D.; Jeudy, V.; Moore, T. A.; Marrows, C. H. Domain-wall motion and interfacial Dzyaloshinskii-Moriya interactions in Pt / Co / Ir (t_{Ir}) / Ta Multilayers. *Phys. Rev. B* **2019**, *99*, 094409.

(12) Emori, S.; Bauer, U.; Ahn, S.-M.; Martinez, E.; Beach, G. S. D. Current-driven dynamics of chiral ferromagnetic domain walls. *Nat. Mater.* **2013**, *12*, 611.

(13) Moreau-Luchaire, C.; Moutafis, C.; Reyren, N.; Sampaio, J.; Vaz, C. A. F.; Van Horne, N.; Bouzehouane, K.; Garcia, K.; Deranlot, C.; Warnicke, P.; Wohlhüter, P.; George, J.-M.; Weigand, M.; Raabe, J.; Cros, V.; Fert, A. Additive interfacial chiral interaction in multilayers for stabilization of small individual skyrmions at room temperature. *Nat. Nanotechnol.* **2016**, *11*, 444.

(14) Yang, H.; Thiaville, A.; Rohart, S.; Fert, A.; Chshiev, M. Anatomy of Dzyaloshinskii-Moriya interaction at Co / Pt Interfaces. *Phys. Rev. Lett.* **2015**, *115*, 267210.

(15) Hahn, E. L. Spin echoes. *Phys. Rev.* **1950**, *80*, 580–594.

(16) Hanson, R.; Dobrovitski, V. V.; Feiguin, A. E.; Gywat, O.; Awschalom, D. D. Coherent dynamics of a single spin interacting with an adjustable spin bath. *Science* **2008**, *32*, 352–

355.
(17) Flebus, B.; Tserkovnyak, Y. Entangling distant spin qubits via a magnetic domain wall. *Phys. Rev. B* **2019**, *99*, 140403.
(18) Zhang, S.; Tchernyshyov, O. Ferromagnetic domain wall as a nonreciprocal string. *Phys. Rev. B* **2018**, *98*, 104411.
(19) Trifunovic, L.; Pedrocchi, F. L.; Loss, D. Long-distance entanglement of spin qubits via ferromagnet. *Phys. Rev. X* **2013**, *3*, 041023.
(20) Flebus, B.; Ochoa, H.; Upadhyaya, P.; Tserkovnyak, Y. Proposal for dynamic imaging of antiferromagnetic domain wall via quantum-impurity relaxometry. *Phys. Rev. B* **2018**, *98*, 180409.
(21) Du, C.; van der Sar, T.; Zhou, T. X.; Upadhyaya, P.; Casola, F.; Zhang, H.; Onbasli, M. C.; Ross, C. A.; Walsworth, R. L.; Tserkovnyak, Y.; Yacoby, A. Control and local measurement of the spin chemical potential in a magnetic insulator. *Science* **2017**, *357* (6347), 195–198.
(22) van der Sar, T.; Casola, F.; Walsworth, R.; Yacoby, A. Nanometre-scale probing of spin waves using single electron spins. *Nat. Commun.* **2015**, *6* (1), 7886.
(23) Kim, S. K.; Tchernyshyov, O.; Galitski, V.; Tserkovnyak, Y. Magnon-induced non-markovian friction of a domain wall in a ferromagnet. *Phys. Rev. B* **2018**, *97*, 174433.
(24) Fang, H.; Zhang, S.; Tserkovnyak, Y. Generalized model of magnon kinetics and subgap magnetic noise. *Phys. Rev. B* **2022**, *105*, 184406.
(25) Flebus, B.; Tserkovnyak, Y. Quantum-impurity relaxometry of magnetization dynamics. *Phys. Rev. Lett.* **2018**, *121*, 187204.
(26) Zou, J.; Zhang, S.; Tserkovnyak, Y. Bell-state generation for spin qubits via dissipative coupling. *Phys. Rev. B* **2022**, *106*, L180406.
(27) Borovik, E.; Kuleshov, V. S. Effective equation of motion of domain walls in a ferromagnet. *Zh. Eksp. Teor. Fiz* **1975**, *68*, 2236.
(28) Tchernyshyov, O. Conserved momenta of a ferromagnetic soliton. *Ann. Phys.* **2015**, *363*, 98–113.
(29) Kubo, R. The fluctuation-dissipation theorem. *Rep. Prog. Phys.* **1966**, *29*, 255.

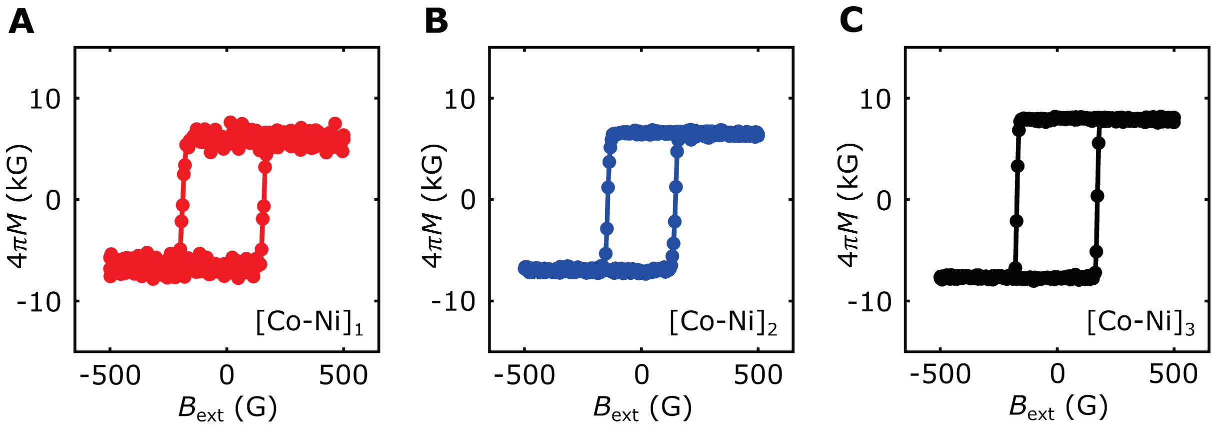

Figure S1. Magnetic characterizations of Co-Ni multilayer samples. Field-dependent magnetization of $[Co\text{-}Ni]_1$ (A), $[Co\text{-}Ni]_2$ (B), and $[Co\text{-}Ni]_3$ (C) thin films measured at room temperature. The external magnetic field B_{ext} is applied along the out-of-plane direction of the film.

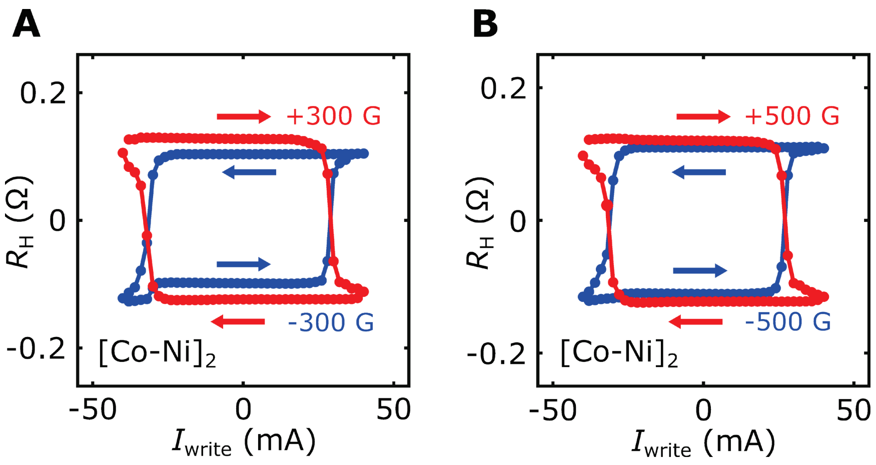

Figure S2. SOT-driven deterministic magnetic switching of a Co-Ni based multilayer device. Anomalous Hall resistance R_H of a patterned Pt/[Co-Ni]$_2$/Ta Hall device measured as a function of the write current I_{write} with application of a longitudinal bias field B_{ext} of ±300 G (A) and ±500 G (B).

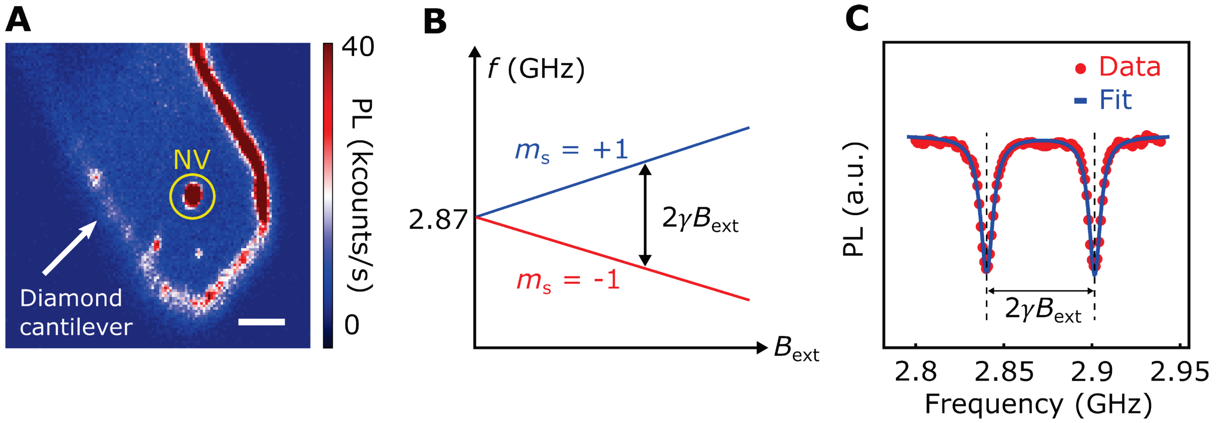

Figure S3. Diamond cantilever and NV ESR measurements. (A) Photoluminescence image of a patterned diamond cantilever containing a single NV center. The scale bar is 3 µm. (B) NV spin energy as a function of an external magnetic field B_{ext} applied along the NV axis. (C) A typical set of optically detected magnetic resonance (ODMR) spectrum of an NV center contained in the diamond cantilever.

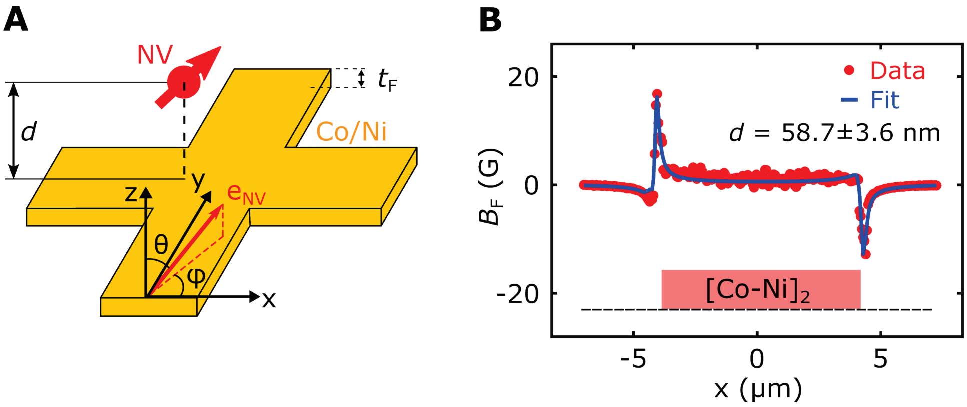

Figure S4. Characterization of NV-to-sample distance. (A) Schematic of the measurement platform and the coordinate system used for numerical analysis. (B) One-dimensional stray B_F measured across the lateral width (x-axis) of a patterned [Co-Ni] Hall device. The NV-to-sample distance can be quantitatively obtained by fitting the spatial distribution of the magnetic stray field to Eq. (3) in the Supplementary Information.

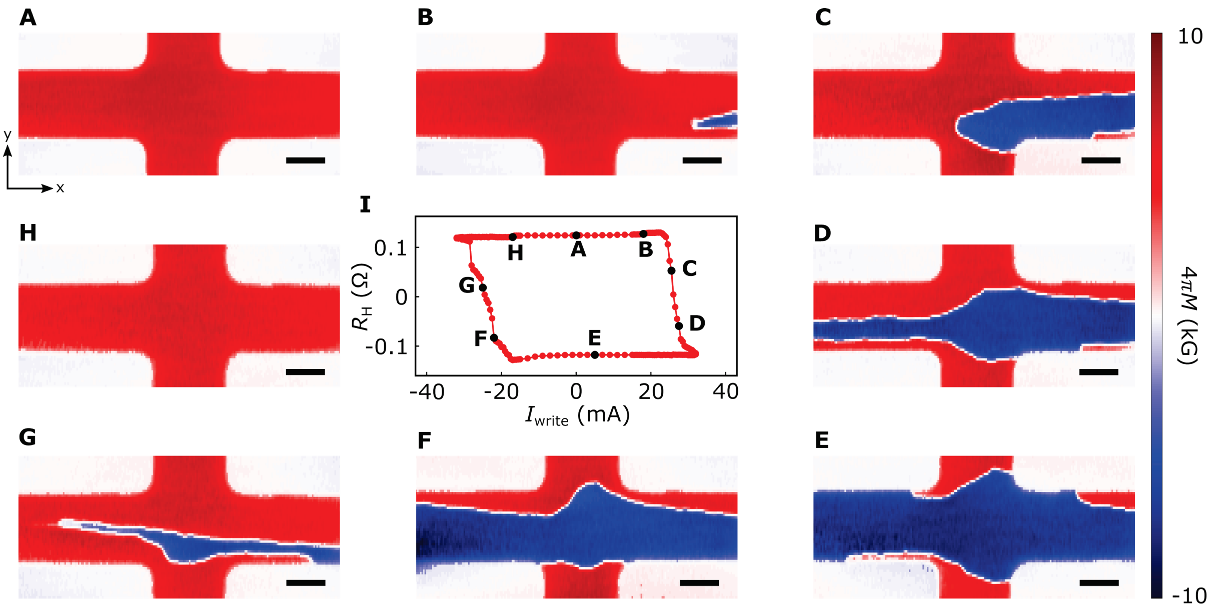

Figure S5. Scanning NV imaging of SOT-driven deterministic magnetic switching. (A-H) Reconstructed magnetization maps of a Pt/[Co-Ni]$_2$/Ta Hall device during the SOT-driven magnetic switching process. The scale bar is 5 μm. (I) Anomalous Hall resistance R_H of the device measured as a function of the write current I_{write} with a longitudinal bias field B_{ext} of ~190 G applied along the current direction. NV images shown in Figures S5A-S5H were performed at the corresponding states from "A" to "H" marked on the current-induced hysteresis loop.

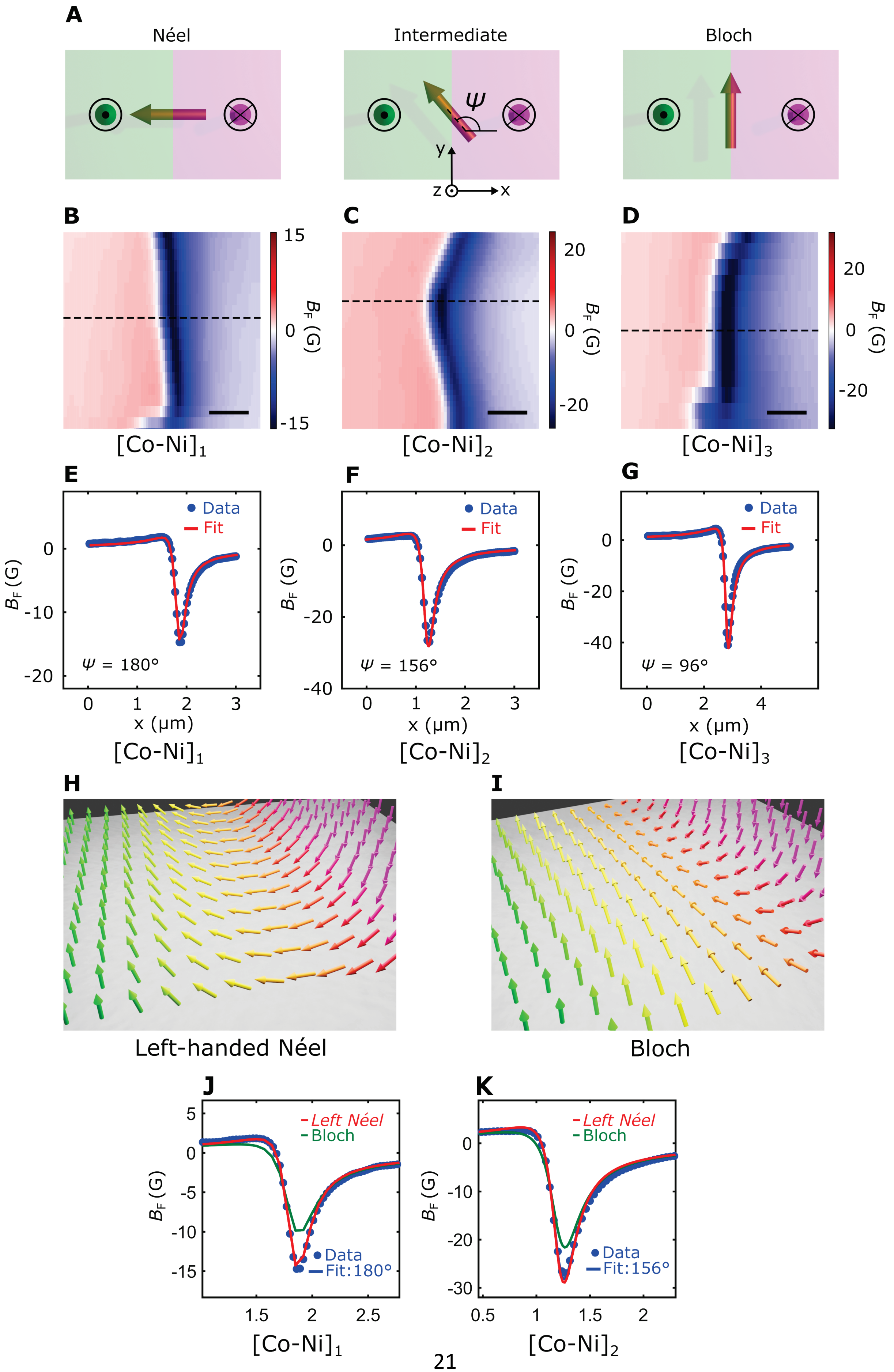

A
Néel
Intermediate
Bloch
ψ
y
z
x
B
C
D
15
0
-15
20
0
-20
20
0
-20
B_F (G)
[Co-Ni]$_1$
[Co-Ni]$_2$
[Co-Ni]$_3$
E
F
G
Data
Fit
ψ = 180°
ψ = 156°
ψ = 96°
B_F (G)
x (μm)
[Co-Ni]$_1$
[Co-Ni]$_2$
[Co-Ni]$_3$
H
I
Left-handed Néel
Bloch
J
K
Left Néel
Bloch
Data
Fit:180°
Fit:156°
B_F (G)
[Co-Ni]$_1$
[Co-Ni]$_2$

Figure S6. Probing the internal spin structure of magnetic domain walls formed in Co-Ni multilayers. (A) Schematics of three types of magnetic domain walls characterized by the in-plane magnetization projection angle ψ. $\psi = 0$, π, and π/2 correspond to the right-handed Néel, left-handed Néel, and Bloch type domain walls, respectively. An intermediate case is characterized by ψ between 0 and π (except π/2). (B-D) Stray field B_F maps generated by magnetic domain walls formed in $[Co/Ni]_1$(B), $[Co/Ni]_2$(C), and $[Co/Ni]_3$(D) multilayer devices. The scale bar is 500 nm. (E-G) One-dimensional profile of magnetic stray field B_F measured along linecuts shown in Figures S6B-S6D. The experimental results are in agreement with the fitting results (red curves), from which the in-plane magnetization projection angle ψ can be quantitatively obtained. (H-I) Schematics of left-handed Néel type (H) and Bloch type (I) magnetic domain walls. (J-K) Comparison between left Néel (180 degree) and Bloch (90 degree) domain walls, over fitting to $[Co-Ni]_1$ (J) and $[Co-Ni]_2$ (K) devices.

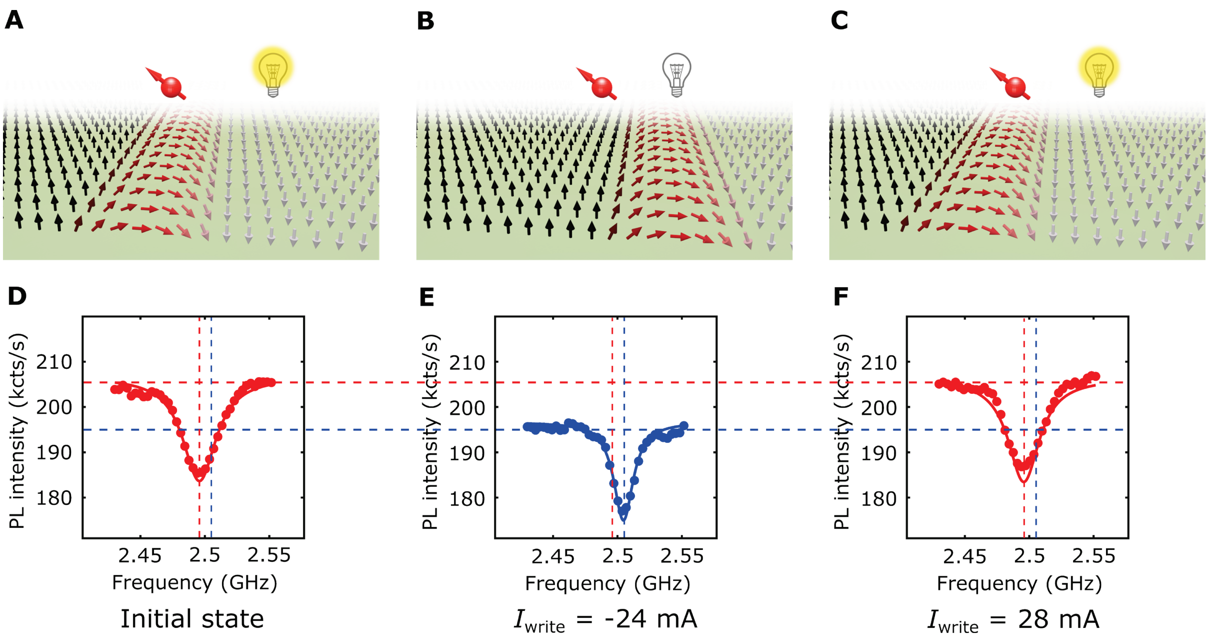

Figure S7. Nanoscale domain wall motion-controlled NV ESR frequencies. (A-C) Schematic illustration of SOT-controlled domain wall motion in relative to a proximate NV center. (D) Measured NV ESR spectrum when a magnetic domain wall is positioned right below the NV spin senor. (E-F) NV ESR spectra recorded when the formed magnetic domain wall is reversibly driven away from (E) and back to (F) the initial position by alternately applying a write current pulse I_{write} of −24 mA and 28 mA, respectively. When retracted from the surface, the NV exhibited photoluminescence of approximately 190 kcounts/s and an ESR frequency of around 2.5 GHz.

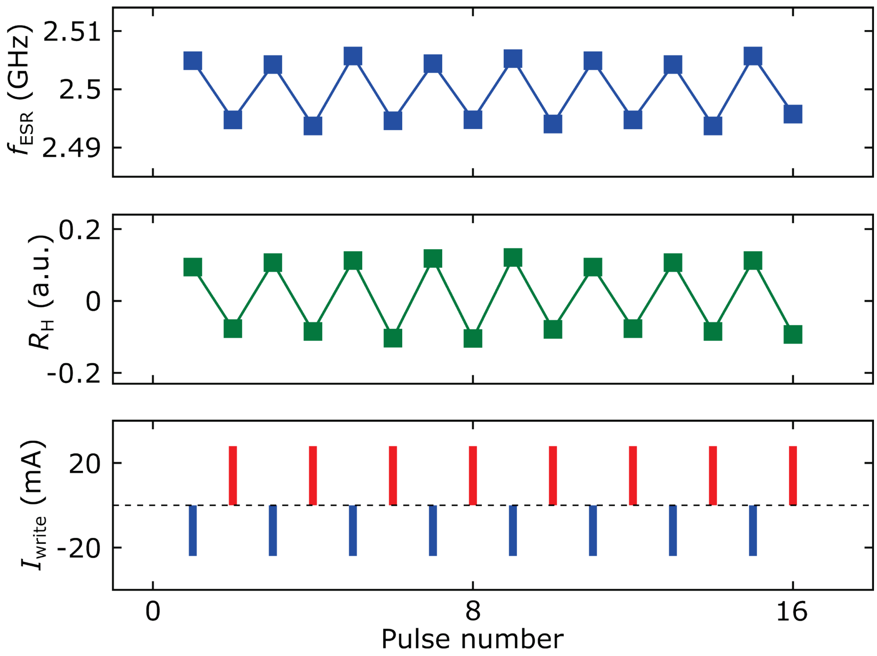

Figure S8. Local control and measurement of NV ESR energies. Switching of NV ESR energies (top panel) by alternatively applying positive and negative write current pulses (bottom panel). The change of the NV spin property can be correlated to the anomalous Hall response (middle panel) driven by reversible domain wall motion in the Hall cross area of the Co-Ni device.

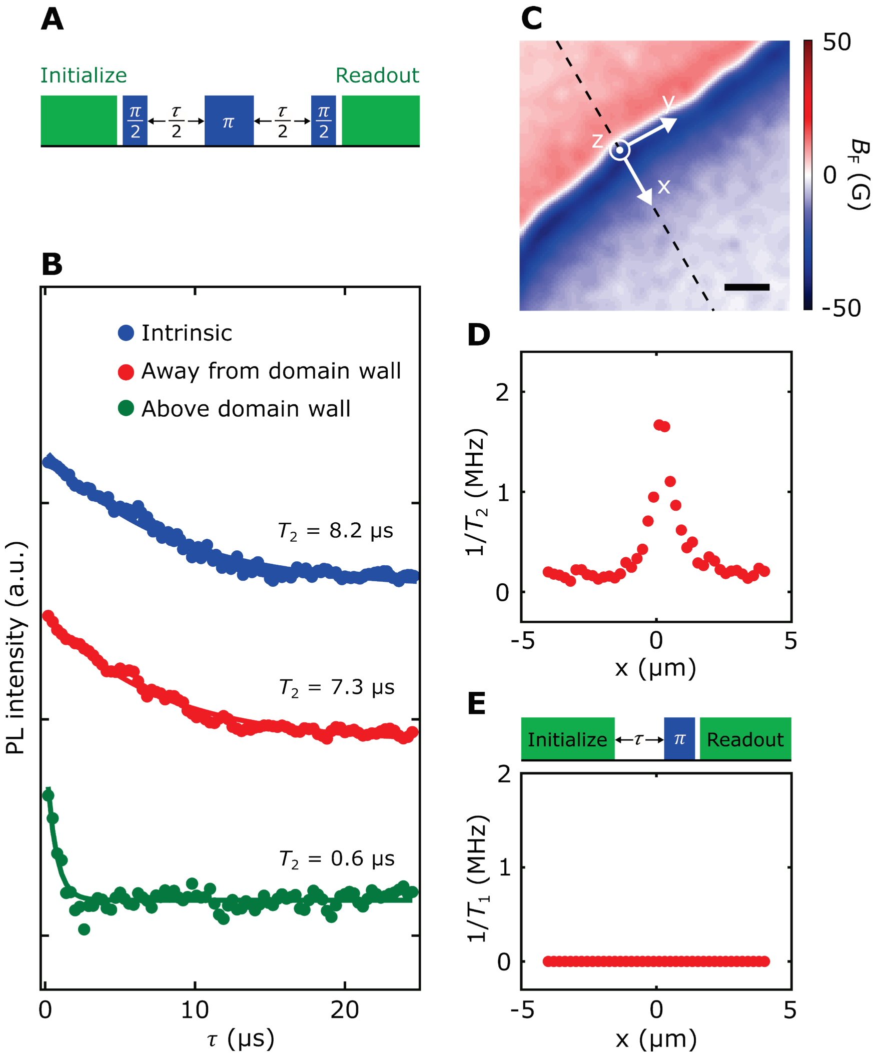

Figure S9. Characterization of nanoscale domain wall motion-controlled NV coherence time. (A) Schematic of the Hanh-echo sequence to measure NV spin coherence time T_2. (B) Time dependence of measured NV PL intensity when the diamond cantilever is positioned right above (green dots) and away from (red dots) a formed magnetic domain wall in the Co-Ni device. The calibrated NV-to-sample distance is ~59 nm in these measurements. Control measurement (blue dots) is also presented to characterize the intrinsic T_2 of the NV center. (C) Scanning NV imaging of stray field B_F arising from a magnetic domain wall in a Co-Ni multilayer device. Scale bar is 200 nm. (D-E) One-dimensional NV spin decoherence rate ($1/T_2$) (D) and relaxation rate ($1/T_1$) (E) measured along the linecut (dashed lines) across the formed magnetic domain wall shown in Figure S9C. The top panel of (E) shows the measurement sequence to characterize the NV spin relaxation time (T_1).

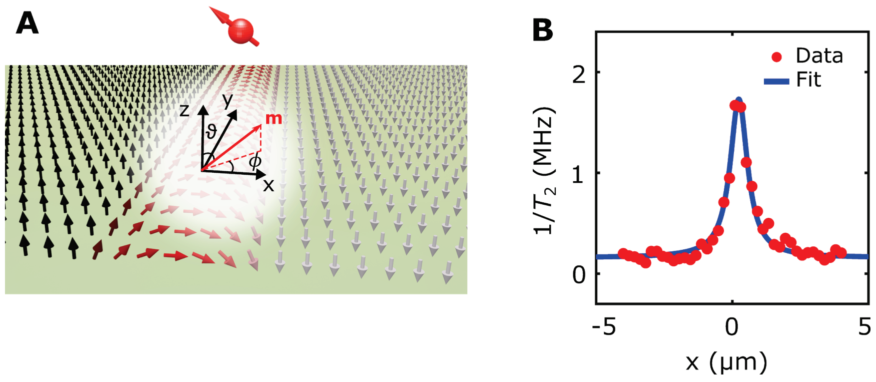

Figure S10. Variation of NV decoherence rate across a ferromagnetic domain wall. (A) Schematic and the coordinate system used for numerical analysis. (B) Experimentally measured one-dimensional NV spin decoherence rate ($1/T_2$) across a magnetic domain wall (red points), in agreement with the theoretical prediction (blue line). The NV spin decoherence rate far from the domain wall is very close to the value when the NV is retracted from the surface, which was approximately 0.125 MHz.
